# Supplementary material for: Healthspan pathway maps in C. elegans and humans highlight transcription, proliferation/biosynthesis and lipids
Source: Aging (Albany NY). 2020 Jul 7;12(13):12534–81. doi: 10.18632/aging.103514 (PMC7377848; doi:10.18632/aging.103514)
Supplement: Supplementary Materials [file aging-12-103514-s001..pdf]

SUPPLEMENTARY MATERIALS

Supplementary Results

Human, largest healthspan pathway, further details

We further investigated the miRNAs that are statistically enriched in the largest healthspan pathway using the TFmir webserver [1]. Notably, hsa-mir-34 stands out as a regulator of the *Notch* genes, and it is implicated in cancer, intracranial aneurysm and heart failure (Supplementary Figure 1, Supplementary Table 9). Additionally, hsa-mir-30 regulates many genes of this healthspan cluster/pathway, including NOTCH2, and it is implicated in the epithelial-mesenchymal transition (EMT), cancer, heart failure and obesity. In fact, the EMT is known to be involved in kidney disease and cancer, mediated by Notch signalling [2–4]. It is also associated with human longevity [5]. Furthermore, miRNA 34 has been associated with schizophrenia [6], and miRNA 34a has been associated with Alzheimer´s Disease [7], and its upregulation was found 30 minutes after fear conditioning in the amygdala, transiently downregulating Notch signaling. Finally, according to Wikipedia's community annotation facilitated by miRBase and Rfam, hsa-mir-34 and hsa-mir-30 are both linked to cancer, see also [8, 9].

Human, second-largest healthspan pathway

The genes in the second-largest pathway/cluster, related to cell proliferation (with links to inflammation and apoptosis) feature downregulation as expected, affecting NFKB1, STAT1, STAT5a and GSK3B, with likely beneficial effects. Specifically, JAK/STAT pathway inhibition is considered to alleviate the cellular senescence-associated secretory phenotype and frailty in old age [10]. Furthermore, [11] demonstrated that the hypothalamus is important for the development of whole-body ageing in mice, and that the underlying basis involves hypothalamic immunity mediated by IKBKB (IkB kinase-b, IKK-b) and NFKB. Zhang et al. developed several interventional models and could show that ageing retardation and lifespan extension were achieved in mice by preventing ageing-related hypothalamic or brain IKBKB and NFKB activation. Further mechanistic studies revealed that IKBKB and NFKB inhibit gonadotropin-releasing hormone (GnRH) to mediate ageing-related hypothalamic GnRH decline, and GnRH treatment amends ageing-impaired neurogenesis and decelerates ageing. For the second-largest pathway, a miRNA enrichment analysis by TFmir highlights hsa-mir-146a, which interacts with NFKB1 and STAT1 in particular, and is implicated in many immunity-related diseases (Supplementary Figure 2, Supplementary Table 10). According to Wikipedia's community annotation, miR-146 is primarily involved in the regulation of inflammation and other processes related to the innate immune system, see also [12].

Human, third-largest healthspan pathway

The third-largest healthspan pathway/cluster features the strong downregulation of APOE, and to a lesser extent also of APOC1. The APO family proteins are all lipid transporters, and severe decreases are detrimental, as they lead to hypercholesterinemia [13]. On the other hand, APOE4 has been widely implicated in the formation of amyloid plaques in Alzheimer's Disease [14], and experimental downregulation showed a protective effect in an Alzheimer Disease mouse model [15]. A supplementary interpretation of the CR-related downregulation found for this healthspan pathway is that fasting reduces lipid load, and hence induces a downregulation of the corresponding transporter proteins.

Human, further gene expression data mapping

The three largest healthspan clusters/pathways were further investigated by mapping aging- and disease-related gene expression data onto them, as published or collected by [16], see Supplementary Methods. In the largest (Notch-related) healthspan pathway (see Supplementary Figure 3), gene expression changes in aging blood clearly show the expected upregulation of Notch genes and LRP1, and the same holds for skin except for Notch3. In the second-largest (proliferation-related) healthspan pathway (see Supplementary Figure 4), most genes are upregulated as expected; again, the signal is stronger in blood than in skin. Finally, the downregulation of lipid-associated genes by CR we observed in the third pathway (see Supplementary Figure 5) is matched by an upregulation of all 4 genes in blood as well as in skin, with the single exception of APOE in skin, the downregulation of which may impair wound healing, since it does so in mice [17]. Furthermore, we mapped disease-related gene expression changes onto the healthspan pathway map, including one cancer entity (pancreatic cancer), coronary disease and Alzheimer disease (AD), see Supplementary Figure 6. We found the genes in the Notch-related healthspan pathway upregulated most consistently in case of Alzheimer disease, whereas genes in the proliferation-related and the lipid-related healthspan pathways were upregulated most consistently in coronary disease. All three healthspan pathways discussed here consist mostly of genes that

are, for higher values of gene expression, affecting health in negative ways; they are mostly downregulated by CR and upregulated by aging and disease.

C. elegans, next-largest healthspan pathways based on genetic/intervention data

In case of the mitochondrial and the hormone response cluster, the rapamycin-induced gene expression changes are not discussed since these are weak; in the *dauer*/dormancy cluster, the daf-16 inhibitor hcf-1 [18] is the most strongly downregulated gene. In the regulation cluster, the strongest changes consist of the downregulation of heat shock response genes hsp-16-41 and hsp-12-3.

C. elegans, next-largest healthspan pathways based on _WormBase_ gene expression effect of intervention data

The ER/peroxisome-related pathway features upregulation of phy-2, daf-22 and acox-1. Specifically, phy-2 is essential for survival and embryonic development [19], while daf-22 catalyzes the final step in the peroxisomal β-oxidation pathway and is essential for *dauer* pheromone production [20], whereas acox-1 is essential for the prevention of fat accumulation [21]. The correlation of fat content and health maintenance was described several times: Fat accumulation in *C. elegans* was found to be decreased in phytochemically treated healthy nematodes [22] [23], during life-prolonging CR [24], or during increased autophagy [25]. Moreover, *ectopic* fat deposition is found in ageing worms and is discussed as a cause of ageing itself [26]. On the other hand, increased fat content was described in long-lived daf-2 mutants [27] and in germline ablated nematodes [28]. Moreover, acox-5 (aka drd-51), which is implicated in starvation-sensing and is downregulated by dietary restriction [29], is downregulated by rapamycin as well. The lysosome-related pathway is dominated by upregulated genes involved in fertility/development (gsp-3, gsp-4, frk-1 and spe-1). Finally, the six genes in the cluster related to morphogenesis are all upregulated (unc-52, involved in neuron differentiation [30] and muscle development [31], is upregulated the strongest), whereas the six genes in the cluster related to biosynthesis and transcription are all downregulated by rapamycin.

Overlap of healthspan pathways, first network alignment (Figure 5, left)

Notably, the serine/tyrosine kinases involved in the alignment are all known to be involved in proliferative processes, albeit in complex ways. The five kinases highlighted by our analysis include pro- and anti-proliferative genes, that is, tumor drivers as well as tumor suppressors. Control of proliferation is arguably the most important aspect of staying healthy, enabling stem cells to perform their function, while avoiding cancer. Accordingly, the independent expression data based on caloric restriction/rapamycin intervention data reflect that proliferation/biosynthesis is generally, but not completely, going down by pro-longevity interventions. Naturally, the phosphorylation status of these kinases would be more informative than their expression at the transcript level. Also, not much is known about the role of PAK4, BRSK2 and MELK in human health or aging. PAK4 is considered to protect cells from apoptosis [32], and a positive role in supporting stem cells is possible [33]. In context of cancer, however, upregulation of PAK4 has been associated with high-grade human breast cancer [34] and with malignancy in a variety of cancer cell lines [35], [36]. PAK4 can positively mediate cell survival and proliferation as well as enhance cell migration and invasion [35], [37], [38]. The inhibition of PAK4 reduced cell proliferation, migration and invasion of gastric cancer cells [35]. Further, depletion of PAK4 is considered to increase cell adhesion dynamics in breast cancer cells; due to its RhoU stabilizing function, it promotes the focal adhesion disassembly via phosphorylation of paxillin [34]. Furthermore, PAK4 modulates Wnt signaling by ß-Catenin regulation, increasing cell proliferation. Concordantly, the Wnt signaling pathway itself promotes intrinsic processes such as cell migration, hematopoiesis and cell polarity, and organogenesis during embryonic development [39], [40] . Concerning BRSK2, which is usually expressed in brain, testis and pancreatic tissue, an enhanced activity in response to DNA damage was reported [41], [42], [43]. In brain, BRSK2 is significant for proper regulation and formation of neuronal polarity in the developing nervous system [43], [44]. Finally, MELK as a stem cell marker is expressed in several types of progenitor cells and hematopoietic stem cells, and it plays key roles in cell cycle, embryonic development and in other crucial cellular processes [45] supporting stem cell function. In turn, upregulation of MELK has been associated with tumor progenitor cells of different origin and direct knock down of MELK leads to significant apoptosis induction [46]. In general, MELK is preferentially upregulated in cancer [47]. Overall, the overlap described highlights a cluster of genes held together mostly by shared protein domains in both species, with alternating evidence for their relation to health.

Overlap of healthspan pathways, second network alignment (Figure 5, right)

In the second alignment, all genes are considered health-related based on the *C. elegans* gene expression data in *WormBase*. The genes acox-1 and daf-22 are involved in the ER, peroxisome and microbody health

cluster (see above), whereas the genes cat-4 and pept-1 were found to be related to ion transport and homeostasis (in a smaller cluster of the original healthspan pathway map, Figure 3). All four genes are differentially regulated in a long-lived sir-2.1 overexpression strain [48] and in nematodes suffering from down-regulation of nhr-49, a key regulator of fat metabolism [49]. Moreover, differential translational regulation of cat-4, pept-1, acox-1, and daf-22 was observed in wildtypes during osmotic stress [50], underlining their role in health- and lifespan regulation. Interactions in *C. elegans* are all based on co-expression, while in human they are based on co-expression, co-localization and physical interaction (except for the interaction of SLC5A1 and GCH1, which is genetic). The independent gene expression data describing the effect of rapamycin in *C. elegans* are plausible for the ER/peroxisome genes acox-1 and daf-22 (see above). For cat-4, no data related to its role in health or survival is available, though the gene is involved in dopamine biosynthetic processes and is a target of the transcription regulator skn-1, which is known to be indispensable for proper stress response [51]. Finally, healthspan-promoting treatments like tannic acid [52], colistin exposure [53], or life-prolonging fasting [54] were shown to induce pept-1 transcription. The expression data in case of human, reflecting caloric restriction effects, are matching expectations (SCP2), are not available (SLC15A1), or are of unknown significance (ACOX1, GCH1; see also [55]). Overall, the overlap described here highlights a cluster of genes held together mostly by co-expression in both species, with a demonstrated relation to health in *C. elegans* only.

Supplementary Discussion

Biological interpretation of the lack of evolutionary conservation

In some sense, the lack of overlap between healthspan pathways in *C. elegans* and humans should not be surprising, and relates to our definition of health as the absence of undesirable conditions (that is, disease and dysfunction). Biologically speaking, each such undesirable condition may have its own etiology, or may partially share an etiology with others, such that depending on environmental factors the prevalence may vary greatly. For example, heart disease appears to be largely absent in Tsimane hunter-gatherers [56], but is a major cause of mortality in modern societies. Any heart disease pathway would thus have a major impact on healthspan in modern societies, but not in the Tsimane. Similar challenges apply to the comparison of healthspan pathways across modern populations as well [57]. It is thus to be expected that healthspan pathways will differ not just across distantly related species, but also among populations of a given species, depending on the environmental factors that push some pathways to more or less important roles in determining healthspan.

Of course it is still possible that there are shared healthspan pathways that operate across populations and species. Indeed, the conservation of genetic pathways related to aging (mTOR, sirtuins, insulin signaling, etc.) [58], [59] strongly implies the existence of shared healthspan pathways, since it is expected that these known aging pathways are also healthspan pathways. The more interesting question is thus whether there might be conserved healthspan pathways that are not also lifespan pathways: pathways that affect health much more than survival. The preliminary answer from this study is that there are few, if any, though we must consider that variation in causes of healthspan deterioration across populations and species might hide some more subtle effects.

From an evolutionary perspective, the question is how selection might act to create and maintain pathways that regulate healthspan. In the case of lifespan, it has been suggested that conserved pathways regulate a mechanism to allow individuals to put reproduction on hold during lean times, increasing lifespan at a cost to reproduction (a “trade-off”), and leading to diverse downstream mechanisms of aging with a shared control switch [60]. One possibility is that healthspan might undergo a similar trade-off, with individuals sacrificing reproduction in order to maintain health, or vice versa, though there is not yet evidence one way or another. If such a trade-off were facultative (i.e., regulated within the lifespan of an individual), we should see variation in gene expression across individuals even in the absence of allelic variation. If it were an obligate trade-off, we might see allelic variation in healthspan pathways. Allelic variation in healthspan could thus either imply (a) that there is some unknown benefit, through the trade-off, to having a shorter healthspan; or (b) that the population is not at evolutionary equilibrium, i.e. is in an environment for which healthspan regulation has not been optimized [61].

Supplementary Methods

Gene sets associated with health, based on WormBase differentially expressed genes

The *basic search* for *expression clusters* in WormBase (http://www.wormbase.org/species/c_elegans/expression_cluster#1-0-5) was used (status: 13th December 2017), to find transcriptomic data for healthspan-promoting compounds in *C. elegans*. For this purpose,

the search term "treated OR treatment OR exposure" was used, which resulted in a total of 323 expression clusters comprising about 100 different chemical, physical, and biological treatments of various kinds (differing by exposure time or dosage, and including RNAi treatment). In order to focus on small molecules, only studies with RNAi-untreated wild type animals were selected. The treatment had to lead to at least one enhanced health-related endpoint such as stress resistance or locomotion. The data sets covered the following substances (with results each described in an accompanying WormBase paper): Allantoin (WBPaper 00048989), astaxanthin (WBPaper00049979), cocoa-peptide 13L (WBPaper00042404), colistin (WBPaper 00045673), 2-deoxy-D-glucose (WBPaper00044434 and WBPaper00031060), garlic extract (WBPaper00046741), hydrogen sulfide (WBPaper00040285), lithium (WBPaper00046415), quercetin (WBPaper00040963), rapamycin (WBPaper00048989), resveratrol (WBPaper00026929), rifampicin (WBPaper00046496), and tannic acid (WBPaper00040963). All differentially expressed genes (DEGs) in the selected gene expression studies were compiled and duplicates were deleted, resulting in 11312 genes that are mentioned in at least one data set. For all genes annotated to at least one GO term (based on the ontology browser in WormBase), the exact number of associated GO terms was determined, by entering all 7646 genes in the search field of the "MGI Gene Ontology Term Finder" (http://www.informatics.jax.org/gotools/MGI_Term_Finder.html). The number of GO terms per gene was counted, and the count of each gene in all DEG lists (regulated, up-regulated only or down-regulated only, respectively) was determined. Finally, all genes were chosen which appear in at least four DEG lists in total or in at least three lists of up-regulated DEGs or in at least three lists of down-regulated DEGs, and which are annotated to at least 14 GO terms. These filter criteria were used to yield a manageable number of annotated genes; the resulting list of 58 genes was then used further. For acox-1.1 and acox-1.5, their alternative nomenclature names acox-1 and acox-5 were used.

Overlaying of expression data onto pathway maps

We searched the GEO (Gene Expression Omnibus) database in December 2017 for datasets/series where effects on healthspan or healthy aging in human or *C. elegans* were actually observed following an intervention. We found two gene expression series describing the effects of caloric restriction, or its mimetic rapamycin, that featured at least 3 replicates, as follows. For *C. elegans,* from GSE64336, "Expression data of worms under different caloric restriction mimetic treatments", we selected 1) wild type versus 2) rapamycin treatment, since the accompanying paper [62] claimed the largest number of differentially expressed genes for this compound (in comparison to the other compound tested, allantoin). For humans, from GSE38012, we selected all 25 samples, 1) Western diet versus 2) caloric restriction diet (for both series, we checked the box plots but we found no outlier distribution of expression values for any sample). We then used the GEO2R tool [63] to compute fold-changes using default parameters, downloaded the resulting tables, imported these into Excel (using "Text" column format for the gene names), removed genes with logFC equal to NaN and sorted, smallest to largest, by absolute fold change so that for genes with more than one probe, the probe with the largest fold-change is taken when the table is imported using Cytoscape. Selecting the "Gene.symbol" column as key column of the table and selecting the "gene name" column created by GeneMANIA as the "Key column for network", we established matching gene names (in case-insensitive mode) in the GEO2R and GeneMANIA tables as the common reference, to then import the tables into Cytoscape. Finally, we adjusted the "Style" of the resulting networks so that the logFC values from GEO2R are mapped continuously to a yellow-blue color scale with the appropriate max/min settings, adding a handle to map a logFC of 0 to white.

Further, we took [16] as reference publication for aging- and disease-related datasets. We took the human aging data published alongside the article, contrasting blood and skin in 24-29 and 45-50 versus 60-65 and 75-80 year-old humans. We took publicly available disease-related datasets listed in Supplementary Table 5 of [16]: for cancer we selected pancreatic cancer (GSE28735) as the only entity with paired data available at GEO; as cardiovascular disease we selected coronary artery disease as the only entity with paired data (taking plaque biopsy data rather than blood), and for neurodegenerative disease, we took Alzheimer Disease data based on brain biopsies. In the latter two cases we chose the tissues directly affected by the respective disease.

Overlap of healthspan pathways

Figure 4 (middle) summarizes the overall approach. The human health-related gene list based on Supplementary Tables 1-3 (Supplementary Table 6) and the health-related gene-expression-based gene list from wormbase (Supplementary Table 8) were investigated jointly. More specifically, both lists were submitted to Wormhole (wormhole.jax.org) on Jan 29, 2018, with "Limit results to ortholog pairs" set to "Do not filter (keep all results)" and with the "Reciprocal best hits (RBHs) only" option, to map from human to *C. elegans* and from *C. elegans* to human, respectively. The two

resulting tables were downloaded, and the ortholog genes were used to obtain two new gene lists: one list consisting of the human health gene list from Supplementary Tables 1-3 supplemented with the human orthologs of the *C. elegans* genes implicated by gene expression in WormBase, and a second list consisting of the health-related gene-expression-based *C. elegans* gene list supplemented with the *C. elegans* orthologs of the human health genes. Both new gene lists were submitted to GeneMANIA with default parameters[1], and the two GeneMANIA reports were exported[2]. From the two GeneMANIA reports, the two interaction networks and the two new lists of genes/nodes in the network were extracted. As input for the GASOLINE network aligner [64], each network was then written to a text file, and a single table of ortholog mappings was created by (a) submitting each of the two new lists of genes/nodes to Wormhole (with parameters as above), converting the "WORMHOLE Score" from 0 (worst) to 1 (best) into an E-value-like score as expected by GASOLINE (using the ad-hoc formula E-Value-substitute=1/WORMHOLE_Score*1E-20, which results in values roughly the same as given in the BLAST-based tables offered by the GASOLINE website as sample input data), and (b) concatenating both Wormhole ortholog tables into a single text file. Submitting the two network files[3] and the single table of ortholog mappings to GASOLINE with default parameters resulted in no alignment of subnetworks, but changing the GASOLINE "density threshold" from 0.8 to 0.5 resulted in the two alignments presented. Finally, the gene expression data describing effects of caloric restriction and of rapamycin were both imported, mapping the expression data to the alignments, and setting node colors, all as described above. Whenever data were processed by Excel, column formats of gene names were set to "Text".

All genes displayed in Supplementary Tables 1-5 and those derived from WormBase gene expression data are summarized in Supplementary Tables 6–8, including GenAge information [65] (https://genomics.senescence.info/genes/; GenAge Build 19; release date: June 24, 2017), if available.

The **web presentation** accompanying this paper employed Cytoscape version 3.6.1 to export the networks and its views as a CytoscapeJS object, employing a library used in version 3.29 (http://js.cytoscape.org) together with an Apache 2 web server [66]. The dynamic highlighting of genes and GO terms in the pathway was implemented in JavaScript. The transcriptional profile of user-selected genes can be inspected in GEO expression data aggregated by the multi-experiment matrix (MEM, https://biit.cs.ut.ee/mem/) [67]. Queried with single genes, the MEM service shows all the transcriptomics experiments of a selected platform and, underneath, all the genes with which the query gene is correlating in its expression [68]. The resulting list is ranked and differences between experiments with respect to the observed correlation are indicated graphically. When queried with a set of genes, specifically with all genes of a healthspan pathway, only correlations of transcripts assigned to these genes are shown. This tell us, in which experiments the genes included in our healthspan pathways interact, and for which conditions there is no concerted action of the healthspan-associated genes. One can thus obtain a characterization of a healthspan pathway in the light of a large set of gene expression experiments.

[1] which ignores some duplications introduced into the lists by a wormhole bug; non-nomenclature gene names for IL-6 and IL-12 were processed correctly.

[2] due to a GeneMANIA feature, reports for the same input gene lists may vary slightly in the last decimal places of some of the scores.

[3] first the C. *elegans* and then the human network; uploading networks the other way around results in slightly different output due to a GASOLINE feature.

Supplementary References

1. Hamed M, Spaniol C, Nazarieh M, Helms V. TFmiR: a web server for constructing and analyzing disease-specific transcription factor and miRNA co-regulatory networks. Nucleic Acids Res. 2015; 43:W283–88. https://doi.org/10.1093/nar/gkv418 PMID:25943543

2. Wang Z, Li Y, Kong D, Sarkar FH. The role of notch signaling pathway in epithelial-mesenchymal transition (EMT) during development and tumor aggressiveness. Curr Drug Targets. 2010; 11:745–51. https://doi.org/10.2174/138945010791170860 PMID:20041844

3. Liu Y. New insights into epithelial-mesenchymal transition in kidney fibrosis. J Am Soc Nephrol. 2010; 21:212–22. https://doi.org/10.1681/ASN.2008121226 PMID:20019167

4. Sharma S, Sirin Y, Susztak K. The story of notch and chronic kidney disease. Curr Opin Nephrol Hypertens. 2011; 20:56–61. https://doi.org/10.1097/MNH.0b013e3283414c88 PMID:21088575

5. ElSharawy A, Keller A, Flachsbart F, Wendschlag A, Jacobs G, Kefer N, Brefort T, Leidinger P, Backes C, Meese E, Schreiber S, Rosenstiel P, Franke A, Nebel A. Genome-wide miRNA signatures of human longevity. Aging Cell. 2012; 11:607–16. https://doi.org/10.1111/j.1474-9726.2012.00824.x PMID:22533606

6. Lai CY, Yu SL, Hsieh MH, Chen CH, Chen HY, Wen CC, Huang YH, Hsiao PC, Hsiao CK, Liu CM, Yang PC, Hwu HG, Chen WJ. MicroRNA expression aberration as potential peripheral blood biomarkers for schizophrenia. PLoS One. 2011; 6:e21635. https://doi.org/10.1371/journal.pone.0021635 PMID:21738743

7. Kiko T, Nakagawa K, Tsuduki T, Furukawa K, Arai H, Miyazawa T. MicroRNAs in plasma and cerebrospinal fluid as potential markers for Alzheimer's disease. J Alzheimers Dis. 2014; 39:253–59. https://doi.org/10.3233/JAD-130932 PMID:24157723

8. Yamakuchi M, Ferlito M, Lowenstein CJ. miR-34a repression of SIRT1 regulates apoptosis. Proc Natl Acad Sci USA. 2008; 105:13421–26. https://doi.org/10.1073/pnas.0801613105 PMID:18755897

9. Li J, Donath S, Li Y, Qin D, Prabhakar BS, Li P. miR-30 regulates mitochondrial fission through targeting p53 and the dynamin-related protein-1 pathway. PLoS Genet. 2010; 6:e1000795. https://doi.org/10.1371/journal.pgen.1000795 PMID:20062521

10. Xu M, Tchkonia T, Ding H, Ogrodnik M, Lubbers ER, Pirtskhalava T, White TA, Johnson KO, Stout MB, Mezera V, Giorgadze N, Jensen MD, LeBrasseur NK, Kirkland JL. JAK inhibition alleviates the cellular senescence-associated secretory phenotype and frailty in old age. Proc Natl Acad Sci USA. 2015; 112:E6301–10. https://doi.org/10.1073/pnas.1515386112 PMID:26578790

11. Zhang G, Li J, Purkayastha S, Tang Y, Zhang H, Yin Y, Li B, Liu G, Cai D. Hypothalamic programming of systemic ageing involving IKK-β, NF-κB and GnRH. Nature. 2013; 497:211–16. https://doi.org/10.1038/nature12143 PMID:23636330

12. Sheedy FJ, O'Neill LA. Adding fuel to fire: microRNAs as a new class of mediators of inflammation. Ann Rheum Dis. 2008 (Suppl 3); 67:iii50–55. https://doi.org/10.1136/ard.2008.100289 PMID:19022814

13. McNeill E, Channon KM, Greaves DR. Inflammatory cell recruitment in cardiovascular disease: murine models and potential clinical applications. Clin Sci (Lond). 2010; 118:641–55. https://doi.org/10.1042/CS20090488 PMID:20210786

14. Kivipelto M, Helkala EL, Laakso MP, Hänninen T, Hallikainen M, Alhainen K, Iivonen S, Mannermaa A, Tuomilehto J, Nissinen A, Soininen H. Apolipoprotein E epsilon4 allele, elevated midlife total cholesterol level, and high midlife systolic blood pressure are independent risk factors for late-life Alzheimer disease. Ann Intern Med. 2002; 137:149–55. https://doi.org/10.7326/0003-4819-137-3-200208060-00006 PMID:12160362

15. Huynh TV, Liao F, Francis CM, Robinson GO, Serrano JR, Jiang H, Roh J, Finn MB, Sullivan PM, Esparza TJ, Stewart FR, Mahan TE, Ulrich JD, et al. Age-dependent effects of apoE reduction using antisense oligonucleotides in a model of β-amyloidosis. Neuron. 2017; 96:1013–23.e4. https://doi.org/10.1016/j.neuron.2017.11.014 PMID:29216448

16. Aramillo Irizar P, Schäuble S, Esser D, Groth M, Frahm C, Priebe S, Baumgart M, Hartmann N, Marthandan S, Menzel U, Müller J, Schmidt S, Ast V, et al. Transcriptomic alterations during ageing reflect the shift from cancer to degenerative diseases in the elderly. Nat Commun. 2018; 9:327. https://doi.org/10.1038/s41467-017-02395-2 PMID:29382830

17. Gordts SC, Muthuramu I, Amin R, Jacobs F, De Geest B. The impact of lipoproteins on wound healing: topical HDL therapy corrects delayed wound healing in apolipoprotein E deficient mice. Pharmaceuticals (Basel). 2014; 7:419–32. https://doi.org/10.3390/ph7040419 PMID:24705596

18. Li J, Ebata A, Dong Y, Rizki G, Iwata T, Lee SS. Caenorhabditis elegans HCF-1 functions in longevity maintenance as a DAF-16 regulator. PLoS Biol. 2008; 6:e233. https://doi.org/10.1371/journal.pbio.0060233 PMID:18828672

19. Wang C, Saar V, Leung KL, Chen L, Wong G. Human amyloid β peptide and tau co-expression impairs behavior and causes specific gene expression changes in caenorhabditis elegans. Neurobiol Dis. 2018; 109:88–101. https://doi.org/10.1016/j.nbd.2017.10.003 PMID:28982592

20. Butcher RA, Ragains JR, Li W, Ruvkun G, Clardy J, Mak HY. Biosynthesis of the caenorhabditis elegans dauer pheromone. Proc Natl Acad Sci USA. 2009; 106:1875–79. https://doi.org/10.1073/pnas.0810338106 PMID:19174521

21. Zhang J, Bakheet R, Parhar RS, Huang CH, Hussain MM, Pan X, Siddiqui SS, Hashmi S. Regulation of fat storage and reproduction by krüppel-like transcription factor KLF3 and fat-associated genes in caenorhabditis elegans. J Mol Biol. 2011; 411:537–53. https://doi.org/10.1016/j.jmb.2011.06.011 PMID:21704635

22. Zarse K, Bossecker A, Müller-Kuhrt L, Siems K, Hernandez MA, Berendsohn WG, Birringer M, Ristow M. The phytochemical glaucarubinone promotes mitochondrial metabolism, reduces body fat, and extends lifespan of caenorhabditis elegans. Horm Metab Res. 2011; 43:241–43. https://doi.org/10.1055/s-0030-1270524 PMID:21264793

23. Zheng S, Liao S, Zou Y, Qu Z, Shen W, Shi Y. Mulberry leaf polyphenols delay aging and regulate fat metabolism via the germline signaling pathway in caenorhabditis elegans. Age (Dordr). 2014; 36:9719. https://doi.org/10.1007/s11357-014-9719-z PMID:25323576

24. Bar DZ, Charar C, Dorfman J, Yadid T, Tafforeau L, Lafontaine DL, Gruenbaum Y. Cell size and fat content of dietary-restricted caenorhabditis elegans are regulated by ATX-2, an mTOR repressor. Proc Natl Acad Sci USA. 2016; 113:E4620–29. https://doi.org/10.1073/pnas.1512156113 PMID:27457958

25. Schiavi A, Torgovnick A, Kell A, Megalou E, Castelein N, Guccini I, Marzocchella L, Gelino S, Hansen M, Malisan F, Condò I, Bei R, Rea SL, et al. Autophagy induction extends lifespan and reduces lipid content in response to frataxin silencing in C. Elegans. Exp Gerontol. 2013; 48:191–201. https://doi.org/10.1016/j.exger.2012.12.002 PMID:23247094

26. Ackerman D, Gems D. The mystery of C. Elegans aging: an emerging role for fat. Distant parallels between C. Elegans aging and metabolic syndrome? Bioessays. 2012; 34:466–71. https://doi.org/10.1002/bies.201100189 PMID:22371137

27. Perez CL, Van Gilst MR. A 13C isotope labeling strategy reveals the influence of insulin signaling on lipogenesis in C. Elegans. Cell Metab. 2008; 8:266–74. https://doi.org/10.1016/j.cmet.2008.08.007 PMID:18762027

28. O'Rourke EJ, Soukas AA, Carr CE, Ruvkun G. C. Elegans major fats are stored in vesicles distinct from lysosome-related organelles. Cell Metab. 2009; 10:430–35. https://doi.org/10.1016/j.cmet.2009.10.002 PMID:19883620

29. Ludewig AH, Klapper M, Döring F. Identifying evolutionarily conserved genes in the dietary restriction response using bioinformatics and subsequent testing in caenorhabditis elegans. Genes Nutr. 2014; 9:363. https://doi.org/10.1007/s12263-013-0363-5 PMID:24311442

30. Díaz-Balzac CA, Lázaro-Peña MI, Tecle E, Gomez N, Bülow HE. Complex cooperative functions of heparan sulfate proteoglycans shape nervous system development in caenorhabditis elegans. G3 (Bethesda). 2014; 4:1859–70. https://doi.org/10.1534/g3.114.012591 PMID:25098771

31. Rogalski TM, Gilchrist EJ, Mullen GP, Moerman DG. Mutations in the unc-52 gene responsible for body wall muscle defects in adult caenorhabditis elegans are located in alternatively spliced exons. Genetics. 1995; 139:159–69. PMID:7535716

32. Gnesutta N, Qu J, Minden A. The serine/threonine kinase PAK4 prevents caspase activation and protects cells from apoptosis. J Biol Chem. 2001; 276:14414–19. https://doi.org/10.1074/jbc.M011046200 PMID:11278822

33. Tyagi N, Marimuthu S, Bhardwaj A, Deshmukh SK, Srivastava SK, Singh AP, McClellan S, Carter JE, Singh S. P-21 activated kinase 4 (PAK4) maintains stem cell-like phenotypes in pancreatic cancer cells through activation of STAT3 signaling. Cancer Lett. 2016; 370:260–67. https://doi.org/10.1016/j.canlet.2015.10.028 PMID:26546043

34. Dart AE, Box GM, Court W, Gale ME, Brown JP, Pinder SE, Eccles SA, Wells CM. PAK4 promotes kinase-independent stabilization of RhoU to modulate cell adhesion. J Cell Biol. 2015; 211:863–79. https://doi.org/10.1083/jcb.201501072 PMID:26598620

35. Zhang J, Wang J, Guo Q, Wang Y, Zhou Y, Peng H, Cheng M, Zhao D, Li F. LCH-7749944, a novel and potent p21-activated kinase 4 inhibitor, suppresses proliferation and invasion in human gastric cancer cells. Cancer Lett. 2012; 317:24–32. https://doi.org/10.1016/j.canlet.2011.11.007 PMID:22085492

36. Callow MG, Clairvoyant F, Zhu S, Schryver B, Whyte DB, Bischoff JR, Jallal B, Smeal T. Requirement for PAK4 in the anchorage-independent growth of human cancer cell lines. J Biol Chem. 2002; 277:550–58. https://doi.org/10.1074/jbc.M105732200 PMID:11668177

37. Siu MK, Chan HY, Kong DS, Wong ES, Wong OG, Ngan HY, Tam KF, Zhang H, Li Z, Chan QK, Tsao SW, Strömblad S, Cheung AN. P21-activated kinase 4 regulates ovarian cancer cell proliferation, migration, and invasion and contributes to poor prognosis in patients. Proc Natl Acad Sci USA. 2010; 107:18622–27. https://doi.org/10.1073/pnas.0907481107 PMID:20926745

38. Ahmed T, Shea K, Masters JR, Jones GE, Wells CM. A PAK4-LIMK1 pathway drives prostate cancer cell migration downstream of HGF. Cell Signal. 2008; 20:1320–28. https://doi.org/10.1016/j.cellsig.2008.02.021 PMID:18424072

39. Chaker M, Minden A, Chen S, Weiss RH, Chini EN, Mahipal A, Azmi AS. Rho GTPase effectors and NAD metabolism in cancer immune suppression. Expert Opin Ther Targets. 2018; 22:9–17. https://doi.org/10.1080/14728222.2018.1413091 PMID:29207896

40. Komiya Y, Habas R. Wnt signal transduction pathways. Organogenesis. 2008; 4:68–75. https://doi.org/10.4161/org.4.2.5851 PMID:19279717

41. Seoighe C, Scally A. Inference of candidate germline mutator loci in humans from genome-wide haplotype data. PLoS Genet. 2017; 13:e1006549. https://doi.org/10.1371/journal.pgen.1006549 PMID:28095480

42. Wang Y, Wan B, Li D, Zhou J, Li R, Bai M, Chen F, Yu L. BRSK2 is regulated by ER stress in protein level and involved in ER stress-induced apoptosis. Biochem Biophys Res Commun. 2012; 423:813–18. https://doi.org/10.1016/j.bbrc.2012.06.046 PMID:22713462

43. Nie J, Liu X, Lilley BN, Zhang H, Pan YA, Kimball SR, Zhang J, Zhang W, Wang L, Jefferson LS, Sanes JR, Han X, Shi Y. SAD-a kinase controls islet β-cell size and function as a mediator of mTORC1 signaling. Proc Natl Acad Sci USA. 2013; 110:13857–62. https://doi.org/10.1073/pnas.1307698110 PMID:23922392

44. Kishi M, Pan YA, Crump JG, Sanes JR. Mammalian SAD kinases are required for neuronal polarization. Science. 2005; 307:929–32. https://doi.org/10.1126/science.1107403 PMID:15705853

45. Jiang P, Zhang D. Maternal embryonic leucine zipper kinase (MELK): a novel regulator in cell cycle control, embryonic development, and cancer. Int J Mol Sci. 2013; 14:21551–60. https://doi.org/10.3390/ijms141121551 PMID:24185907

46. Giuliano CJ, Lin A, Smith JC, Palladino AC, Sheltzer JM. MELK expression correlates with tumor mitotic activity but is not required for cancer growth. Elife. 2018; 7:e32838. https://doi.org/10.7554/eLife.32838 PMID:29417930

47. Ganguly R, Mohyeldin A, Thiel J, Kornblum HI, Beullens M, Nakano I. MELK-a conserved kinase: functions, signaling, cancer, and controversy. Clin Transl Med. 2015; 4:11. https://doi.org/10.1186/s40169-014-0045-y PMID:25852826

48. Viswanathan M, Guarente L. Regulation of caenorhabditis elegans lifespan by sir-2.1 transgenes. Nature. 2011; 477:E1–2. https://doi.org/10.1038/nature10440 PMID:21938026

49. Van Gilst MR, Hadjivassiliou H, Jolly A, Yamamoto KR. Nuclear hormone receptor NHR-49 controls fat consumption and fatty acid composition in C. Elegans. PLoS Biol. 2005; 3:e53. https://doi.org/10.1371/journal.pbio.0030053 PMID:15719061

50. Burton NO, Furuta T, Webster AK, Kaplan RE, Baugh LR, Arur S, Horvitz HR. Insulin-like signalling to the maternal germline controls progeny response to osmotic stress. Nat Cell Biol. 2017; 19:252–57. https://doi.org/10.1038/ncb3470 PMID:28166192

51. Oliveira RP, Porter Abate J, Dilks K, Landis J, Ashraf J, Murphy CT, Blackwell TK. Condition-adapted stress and longevity gene regulation by caenorhabditis elegans SKN-1/nrf. Aging Cell. 2009; 8:524–41. https://doi.org/10.1111/j.1474-9726.2009.00501.x PMID:19575768

52. Pietsch K, Saul N, Swain SC, Menzel R, Steinberg CE, Stürzenbaum SR. Meta-analysis of global transcriptomics suggests that conserved genetic pathways are responsible for quercetin and tannic acid mediated longevity in C. Elegans. Front Genet. 2012; 3:48. https://doi.org/10.3389/fgene.2012.00048 PMID:22493606

53. Cai Y, Cao X, Aballay A. Whole-animal chemical screen identifies colistin as a new immunomodulator that targets conserved pathways. mBio. 2014; 5:e01235–14. https://doi.org/10.1128/mBio.01235-14 PMID:25118236

54. Ihara A, Uno M, Miyatake K, Honjoh S, Nishida E. Cholesterol regulates DAF-16 nuclear localization and fasting-induced longevity in C. Elegans. Exp Gerontol. 2017; 87:40–47. https://doi.org/10.1016/j.exger.2016.10.011 PMID:27989925

55. Swindell WR. Genes and gene expression modules associated with caloric restriction and aging in the laboratory mouse. BMC Genomics. 2009; 10:585. https://doi.org/10.1186/1471-2164-10-585 PMID:19968875

56. Gurven M, Kaplan H, Winking J, Eid Rodriguez D,

Vasunilashorn S, Kim JK, Finch C, Crimmins E. Inflammation and infection do not promote arterial aging and cardiovascular disease risk factors among lean horticulturalists. PLoS One. 2009; 4:e6590. https://doi.org/10.1371/journal.pone.0006590 PMID:19668697

57. Cohen AA, Legault V, Fuellen G, Fülöp T, Fried LP, Ferrucci L. The risks of biomarker-based epidemiology: associations of circulating calcium levels with age, mortality, and frailty vary substantially across populations. Exp Gerontol. 2018; 107:11–17. https://doi.org/10.1016/j.exger.2017.07.011 PMID:28723411

58. Kapahi P, Chen D, Rogers AN, Katewa SD, Li PW, Thomas EL, Kockel L. With TOR, less is more: a key role for the conserved nutrient-sensing TOR pathway in aging. Cell Metab. 2010; 11:453–65. https://doi.org/10.1016/j.cmet.2010.05.001 PMID:20519118

59. Barbieri M, Bonafè M, Franceschi C, Paolisso G. insulin/IGF-I-signaling pathway: an evolutionarily conserved mechanism of longevity from yeast to humans. Am J Physiol Endocrinol Metab. 2003; 285:E1064–71. https://doi.org/10.1152/ajpendo.00296.2003 PMID:14534077

60. Partridge L, Gems D. Mechanisms of ageing: public or private? Nat Rev Genet. 2002; 3:165–75. https://doi.org/10.1038/nrg753 PMID:11972154

61. Pease C, Lande R, Bull J. A model of population growth, dispersal and evolution in a changing environment. Ecology. 1989; 70:1657–64. https://doi.org/10.2307/1938100

62. Calvert S, Tacutu R, Sharifi S, Teixeira R, Ghosh P, de Magalhães JP. A network pharmacology approach reveals new candidate caloric restriction mimetics in C. Elegans. Aging Cell. 2016; 15:256–66. https://doi.org/10.1111/acel.12432 PMID:26676933

63. Barrett T, Wilhite SE, Ledoux P, Evangelista C, Kim IF, Tomashevsky M, Marshall KA, Phillippy KH, Sherman PM, Holko M, Yefanov A, Lee H, Zhang N, et al. NCBI GEO: archive for functional genomics data sets—update. Nucleic Acids Res. 2013; 41:D991–95. https://doi.org/10.1093/nar/gks1193 PMID:23193258

64. Micale G, Continella A, Ferro A, Giugno R, Pulvirenti A. GASOLINE: a cytoscape app for multiple local alignment of PPI networks. F1000Res. 2014; 3:140. https://doi.org/10.12688/f1000research.4537.2 PMID:25324964

65. Tacutu R, Thornton D, Johnson E, Budovsky A, Barardo D, Craig T, Diana E, Lehmann G, Toren D, Wang J, Fraifeld VE, de Magalhães JP. Human ageing genomic resources: new and updated databases. Nucleic Acids Res. 2018; 46:D1083–90. https://doi.org/10.1093/nar/gkx1042 PMID:29121237

66. Franz M, Lopes CT, Huck G, Dong Y, Sumer O, Bader GD. Cytoscape.js: a graph theory library for visualisation and analysis. Bioinformatics. 2016; 32:309–11. https://doi.org/10.1093/bioinformatics/btv557 PMID:26415722

67. Adler P, Kolde R, Kull M, Tkachenko A, Peterson H, Reimand J, Vilo J. Mining for coexpression across hundreds of datasets using novel rank aggregation and visualization methods. Genome Biol. 2009; 10:R139. https://doi.org/10.1186/gb-2009-10-12-r139 PMID:19961599

68. Kolesnikov N, Hastings E, Keays M, Melnichuk O, Tang YA, Williams E, Dylag M, Kurbatova N, Brandizi M, Burdett T, Megy K, Pilicheva E, Rustici G, et al. ArrayExpress update—simplifying data submissions. Nucleic Acids Res. 2015; 43:D1113–16. https://doi.org/10.1093/nar/gku1057 PMID:25361974

69. Minster RL, Sanders JL, Singh J, Kammerer CM, Barmada MM, Matteini AM, Zhang Q, Wojczynski MK, Daw EW, Brody JA, Arnold AM, Lunetta KL, Murabito JM, et al. Genome-wide association study and linkage analysis of the healthy aging index. J Gerontol A Biol Sci Med Sci. 2015; 70:1003–08. https://doi.org/10.1093/gerona/glv006 PMID:25758594

70. Druley TE, Wang L, Lin SJ, Lee JH, Zhang Q, Daw EW, Abel HJ, Chasnoff SE, Ramos EI, Levinson BT, Thyagarajan B, Newman AB, Christensen K, et al. Candidate gene resequencing to identify rare, pedigree-specific variants influencing healthy aging phenotypes in the long life family study. BMC Geriatr. 2016; 16:80. https://doi.org/10.1186/s12877-016-0253-y PMID:27060904

71. Singh J, Minster RL, Schupf N, Kraja A, Liu Y, Christensen K, Newman AB, Kammerer CM. Genomewide association scan of a mortality associated endophenotype for a long and healthy life in the long life family study. J Gerontol A Biol Sci Med Sci. 2017; 72:1411–16. https://doi.org/10.1093/gerona/glx011 PMID:28329217

72. Mekli K, Marshall A, Nazroo J, Vanhoutte B, Pendleton N. Genetic variant of interleukin-18 gene is associated

with the frailty index in the english longitudinal study of ageing. Age Ageing. 2015; 44:938–42. https://doi.org/10.1093/ageing/afv122 PMID:26396182

73. Ho YY, Matteini AM, Beamer B, Fried L, Xue QL, Arking DE, Chakravarti A, Fallin MD, Walston J. Exploring biologically relevant pathways in frailty. J Gerontol A Biol Sci Med Sci. 2011; 66:975–79. https://doi.org/10.1093/gerona/glr061 PMID:21743092

74. Erikson GA, Bodian DL, Rueda M, Molparia B, Scott ER, Scott-Van Zeeland AA, Topol SE, Wineinger NE, Niederhuber JE, Topol EJ, Torkamani A. Whole-genome sequencing of a healthy aging cohort. Cell. 2016; 165:1002–11. https://doi.org/10.1016/j.cell.2016.03.022 PMID:27114037

75. Christensen K, McGue M. Genetics: healthy ageing, the genome and the environment. Nat Rev Endocrinol. 2016; 12:378–80. https://doi.org/10.1038/nrendo.2016.79 PMID:27230948

76. He L, Kernogitski Y, Kulminskaya I, Loika Y, Arbeev KG, Loiko E, Bagley O, Duan M, Yashkin A, Ukraintseva SV, Kovtun M, Yashin AI, Kulminski AM. Pleiotropic meta-analyses of longitudinal studies discover novel genetic variants associated with age-related diseases. Front Genet. 2016; 7:179. https://doi.org/10.3389/fgene.2016.00179 PMID:27790247

77. Tindale LC, Leach S, Spinelli JJ, Brooks-Wilson AR. Lipid and Alzheimer's disease genes associated with healthy aging and longevity in healthy oldest-old. Oncotarget. 2017; 8:20612–21. https://doi.org/10.18632/oncotarget.15296 PMID:28206976

78. Yao C, Joehanes R, Johnson AD, Huan T, Esko T, Ying S, Freedman JE, Murabito J, Lunetta KL, Metspalu A, Munson PJ, Levy D. Sex- and age-interacting eQTLs in human complex diseases. Hum Mol Genet. 2014; 23:1947–56. https://doi.org/10.1093/hmg/ddt582 PMID:24242183

79. Kauwe JS, Goate A. Genes for a 'Wellderly' life. Trends Mol Med. 2016; 22:637–39. https://doi.org/10.1016/j.molmed.2016.05.011 PMID:27312143

80. Pilling LC, Harries LW, Powell J, Llewellyn DJ, Ferrucci L, Melzer D. Genomics and successful aging: grounds for renewed optimism? J Gerontol A Biol Sci Med Sci. 2012; 67:511–19. https://doi.org/10.1093/gerona/gls091 PMID:22454374

81. Jeck WR, Siebold AP, Sharpless NE. Review: a meta-analysis of GWAS and age-associated diseases. Aging Cell. 2012; 11:727–31. https://doi.org/10.1111/j.1474-9726.2012.00871.x PMID:22888763

82. Fortney K, Dobriban E, Garagnani P, Pirazzini C, Monti D, Mari D, Atzmon G, Barzilai N, Franceschi C, Owen AB, Kim SK. Genome-wide scan informed by age-related disease identifies loci for exceptional human longevity. PLoS Genet. 2015; 11:e1005728. https://doi.org/10.1371/journal.pgen.1005728 PMID:26677855

83. McDaid AF, Joshi PK, Porcu E, Komljenovic A, Li H, Sorrentino V, Litovchenko M, Bevers RP, Rüeger S, Reymond A, Bochud M, Deplancke B, Williams RW, et al. Bayesian association scan reveals loci associated with human lifespan and linked biomarkers. Nat Commun. 2017; 8:15842. https://doi.org/10.1038/ncomms15842 PMID:28748955

84. Iwasa H, Yu S, Xue J, Driscoll M. Novel EGF pathway regulators modulate C. Elegans healthspan and lifespan via EGF receptor, PLC-gamma, and IP3R activation. Aging Cell. 2010; 9:490–505. https://doi.org/10.1111/j.1474-9726.2010.00575.x PMID:20497132

85. Eckers A, Jakob S, Heiss C, Haarmann-Stemmann T, Goy C, Brinkmann V, Cortese-Krott MM, Sansone R, Esser C, Ale-Agha N, Altschmied J, Ventura N, Haendeler J. The aryl hydrocarbon receptor promotes aging phenotypes across species. Sci Rep. 2016; 6:19618. https://doi.org/10.1038/srep19618 PMID:26790370

86. Sutphin GL, Backer G, Sheehan S, Bean S, Corban C, Liu T, Peters MJ, van Meurs JB, Murabito JM, Johnson AD, Korstanje R, Cohorts for Heart and Aging Research in Genomic Epidemiology (CHARGE) Consortium Gene Expression Working Group. Caenorhabditis elegans orthologs of human genes differentially expressed with age are enriched for determinants of longevity. Aging Cell. 2017; 16:672–82. https://doi.org/10.1111/acel.12595 PMID:28401650

87. Leonov A, Arlia-Ciommo A, Piano A, Svistkova V, Lutchman V, Medkour Y, Titorenko VI. Longevity extension by phytochemicals. Molecules. 2015; 20:6544–72. https://doi.org/10.3390/molecules20046544 PMID:25871373

88. Ding AJ, Zheng SQ, Huang XB, Xing TK, Wu GS, Sun HY, Qi SH, Luo HR. Current perspective in the discovery of anti-aging agents from natural products. Nat Prod Bioprospect. 2017; 7:335–404.

https://doi.org/10.1007/s13659-017-0135-9 PMID:28567542

89. Jafari M. Healthspan pharmacology. Rejuvenation Res. 2015; 18:573–80. https://doi.org/10.1089/rej.2015.1774 PMID:26444965

90. Nguyen TT, Caito SW, Zackert WE, West JD, Zhu S, Aschner M, Fessel JP, Roberts LJ 2nd. Scavengers of reactive γ-ketoaldehydes extend caenorhabditis elegans lifespan and healthspan through protein-level interactions with SIR-2.1 and ETS-7. Aging (Albany NY). 2016; 8:1759–80. https://doi.org/10.18632/aging.101011 PMID:27514077

91. Li Y, Shao Y, Tong Y, Shen T, Zhang J, Li Y, Gu H, Li F. Nucleo-cytoplasmic shuttling of PAK4 modulates β-catenin intracellular translocation and signaling. Biochim Biophys Acta. 2012; 1823:465–75. https://doi.org/10.1016/j.bbamcr.2011.11.013 PMID:22173096

92. Abo A, Qu J, Cammarano MS, Dan C, Fritsch A, Baud V, Belisle B, Minden A. PAK4, a novel effector for Cdc42Hs, is implicated in the reorganization of the actin cytoskeleton and in the formation of filopodia. EMBO J. 1998; 17:6527–40. https://doi.org/10.1093/emboj/17.22.6527 PMID:9822598

93. Itakura A, Aslan JE, Kusanto BT, Phillips KG, Porter JE, Newton PK, Nan X, Insall RH, Chernoff J, McCarty OJ. P21-activated kinase (PAK) regulates cytoskeletal reorganization and directional migration in human neutrophils. PLoS One. 2013; 8:e73063. https://doi.org/10.1371/journal.pone.0073063 PMID:24019894

94. Mansfeld J, Urban N, Priebe S, Groth M, Frahm C, Hartmann N, Gebauer J, Ravichandran M, Dommaschk A, Schmeisser S, Kuhlow D, Monajembashi S, Bremer-Streck S, et al. Branched-chain amino acid catabolism is a conserved regulator of physiological ageing. Nat Commun. 2015; 6:10043. https://doi.org/10.1038/ncomms10043 PMID:26620638

95. Lu R, Niida H, Nakanishi M. Human SAD1 kinase is involved in UV-induced DNA damage checkpoint function. J Biol Chem. 2004; 279:31164–70. https://doi.org/10.1074/jbc.M404728200 PMID:15150265

96. Edwards SL, Morrison LM, Yorks RM, Hoover CM, Boominathan S, Miller KG. UNC-16 (JIP3) acts through synapse-assembly proteins to inhibit the active transport of cell soma organelles to caenorhabditis elegans motor neuron axons. Genetics. 2015; 201:117–41. https://doi.org/10.1534/genetics.115.177345 PMID:26354976

97. Hung W, Hwang C, Po MD, Zhen M. Neuronal polarity is regulated by a direct interaction between a scaffolding protein, neurabin, and a presynaptic SAD-1 kinase in caenorhabditis elegans. Development. 2007; 134:237–49. https://doi.org/10.1242/dev.02725 PMID:17151015

98. Kim JS, Hung W, Narbonne P, Roy R, Zhen M. C. Elegans STRADalpha and SAD cooperatively regulate neuronal polarity and synaptic organization. Development. 2010; 137:93–102. https://doi.org/10.1242/dev.041459 PMID:20023164

99. Hirose T, Horvitz HR. An Sp1 transcription factor coordinates caspase-dependent and -independent apoptotic pathways. Nature. 2013; 500:354–58. https://doi.org/10.1038/nature12329 PMID:23851392

100. Offenburger SL, Bensaddek D, Murillo AB, Lamond AI, Gartner A. Comparative genetic, proteomic and phosphoproteomic analysis of C. Elegans embryos with a focus on ham-1/STOX and pig-1/MELK in dopaminergic neuron development. Sci Rep. 2017; 7:4314. https://doi.org/10.1038/s41598-017-04375-4 PMID:28659600

101. Cordes S, Frank CA, Garriga G. The C. Elegans MELK ortholog PIG-1 regulates cell size asymmetry and daughter cell fate in asymmetric neuroblast divisions. Development. 2006; 133:2747–56. https://doi.org/10.1242/dev.02447 PMID:16774992

102. Wu D, Pan W. GSK3: a multifaceted kinase in Wnt signaling. Trends Biochem Sci. 2010; 35:161–68. https://doi.org/10.1016/j.tibs.2009.10.002 PMID:19884009

103. Schaffer BA, Bertram L, Miller BL, Mullin K, Weintraub S, Johnson N, Bigio EH, Mesulam M, Wiedau-Pazos M, Jackson GR, Cummings JL, Cantor RM, Levey AI, et al. Association of GSK3B with Alzheimer disease and frontotemporal dementia. Arch Neurol. 2008; 65:1368–74. https://doi.org/10.1001/archneur.65.10.1368 PMID:18852354

104. Kwok JB, Hallupp M, Loy CT, Chan DK, Woo J, Mellick GD, Buchanan DD, Silburn PA, Halliday GM, Schofield PR. GSK3B polymorphisms alter transcription and splicing in parkinson's disease. Ann Neurol. 2005; 58:829–39. https://doi.org/10.1002/ana.20691 PMID:16315267

105. Thorpe CJ, Schlesinger A, Bowerman B. Wnt signalling in caenorhabditis elegans: regulating repressors and polarizing the cytoskeleton. Trends Cell Biol. 2000; 10:10–17. https://doi.org/10.1016/s0962-8924(99)01672-4 PMID:10603471

106. Kraemer BC, Burgess JK, Chen JH, Thomas JH, Schellenberg GD. Molecular pathways that influence human tau-induced pathology in caenorhabditis elegans. Hum Mol Genet. 2006; 15:1483–96. https://doi.org/10.1093/hmg/ddl067 PMID:16600994

107. Pinto SM, Hengartner MO. Cleaning up the mess: cell corpse clearance in caenorhabditis elegans. Curr Opin Cell Biol. 2012; 24:881–88. https://doi.org/10.1016/j.ceb.2012.11.002 PMID:23206434

108. Hannon GJ, Beach D. p15INK4B is a potential effector of TGF-beta-induced cell cycle arrest. Nature. 1994; 371:257–61. https://doi.org/10.1038/371257a0 PMID:8078588

109. Boström J, Meyer-Puttlitz B, Wolter M, Blaschke B, Weber RG, Lichter P, Ichimura K, Collins VP, Reifenberger G. Alterations of the tumor suppressor genes CDKN2A (p16(INK4a)), p14(ARF), CDKN2B (p15(INK4b)), and CDKN2C (p18(INK4c)) in atypical and anaplastic meningiomas. Am J Pathol. 2001; 159:661–69. https://doi.org/10.1016/S0002-9440(10)61737-3 PMID:11485924

110. Hannou SA, Wouters K, Paumelle R, Staels B. Functional genomics of the CDKN2A/B locus in cardiovascular and metabolic disease: what have we learned from GWASs? Trends Endocrinol Metab. 2015; 26:176–84. https://doi.org/10.1016/j.tem.2015.01.008 PMID:25744911

111. Engelmann I, Griffon A, Tichit L, Montañana-Sanchis F, Wang G, Reinke V, Waterston RH, Hillier LW, Ewbank JJ. A comprehensive analysis of gene expression changes provoked by bacterial and fungal infection in C. Elegans. PLoS One. 2011; 6:e19055. https://doi.org/10.1371/journal.pone.0019055 PMID:21602919

112. Poirier Y, Antonenkov VD, Glumoff T, Hiltunen JK. Peroxisomal beta-oxidation—a metabolic pathway with multiple functions. Biochim Biophys Acta. 2006; 1763:1413–26. https://doi.org/10.1016/j.bbamcr.2006.08.034 PMID:17028011

113. Baarine M, Andréoletti P, Athias A, Nury T, Zarrouk A, Ragot K, Vejux A, Riedinger JM, Kattan Z, Bessede G, Trompier D, Savary S, Cherkaoui-Malki M, Lizard G. Evidence of oxidative stress in very long chain fatty acid—treated oligodendrocytes and potentialization of ROS production using RNA interference-directed knockdown of ABCD1 and ACOX1 peroxisomal proteins. Neuroscience. 2012; 213:1–18. https://doi.org/10.1016/j.neuroscience.2012.03.058 PMID:22521832

114. Joo HJ, Kim KY, Yim YH, Jin YX, Kim H, Kim MY, Paik YK. Contribution of the peroxisomal acox gene to the dynamic balance of daumone production in caenorhabditis elegans. J Biol Chem. 2010; 285:29319–25. https://doi.org/10.1074/jbc.M110.122663 PMID:20610393

115. Filipp FV, Sattler M. Conformational plasticity of the lipid transfer protein SCP2. Biochemistry. 2007; 46:7980–91. https://doi.org/10.1021/bi6025616 PMID:17566986

116. Steinberg S, Raymond G, Braverman N, Moser A. Zellweger Spectrum Disorder. GeneReview. 2017: https://www.ncbi.nlm.nih.gov/books/NBK1448/.

117. Li S, Xu S, Ma Y, Wu S, Feng Y, Cui Q, Chen L, Zhou S, Kong Y, Zhang X, Yu J, Wu M, Zhang SO. A genetic screen for mutants with supersized lipid droplets in caenorhabditis elegans. G3 (Bethesda). 2016; 6:2407–19. https://doi.org/10.1534/g3.116.030866 PMID:27261001

118. Zhang L, Rao F, Zhang K, Khandrika S, Das M, Vaingankar SM, Bao X, Rana BK, Smith DW, Wessel J, Salem RM, Rodriguez-Flores JL, Mahata SK, et al. Discovery of common human genetic variants of GTP cyclohydrolase 1 (GCH1) governing nitric oxide, autonomic activity, and cardiovascular risk. J Clin Invest. 2007; 117:2658–71. https://doi.org/10.1172/JCI31093 PMID:17717598

119. Giri S, Naiya T, Roy S, Das G, Wali GM, Das SK, Ray K, Ray J. A compound heterozygote for GCH1 mutation represents a case of atypical dopa-responsive dystonia. J Mol Neurosci. 2019; 68:214–20. https://doi.org/10.1007/s12031-019-01301-3 PMID:30911941

120. Sawin ER, Ranganathan R, Horvitz HR. C. Elegans locomotory rate is modulated by the environment through a dopaminergic pathway and by experience through a serotonergic pathway. Neuron. 2000; 26:619–31. https://doi.org/10.1016/s0896-6273(00)81199-x PMID:10896158

121. Gonzalez DE, Covitz KM, Sadée W, Mrsny RJ. An oligopeptide transporter is expressed at high levels in the pancreatic carcinoma cell lines AsPc-1 and capan-2. Cancer Res. 1998; 58:519–25. PMID:9458100

122. Spanier B. Transcriptional and functional regulation of the intestinal peptide transporter PEPT1. J Physiol. 2014; 592:871–79. https://doi.org/10.1113/jphysiol.2013.258889 PMID:23959672

123. Meissner B, Boll M, Daniel H, Baumeister R. Deletion of the intestinal peptide transporter affects insulin and TOR signaling in caenorhabditis elegans. J Biol Chem. 2004; 279:36739–45. https://doi.org/10.1074/jbc.M403415200 PMID:15155758
