# Supplementary material for: Healthspan pathway maps in C. elegans and humans highlight transcription, proliferation/biosynthesis and lipids
Source: Aging (Albany NY). 2020 Jul 7;12(13):12534–81. doi: 10.18632/aging.103514 (PMC7377848; doi:10.18632/aging.103514)
Supplement: Supplementary Figures [file aging-12-103514-s003..pdf]

## SUPPLEMENTARY FIGURES

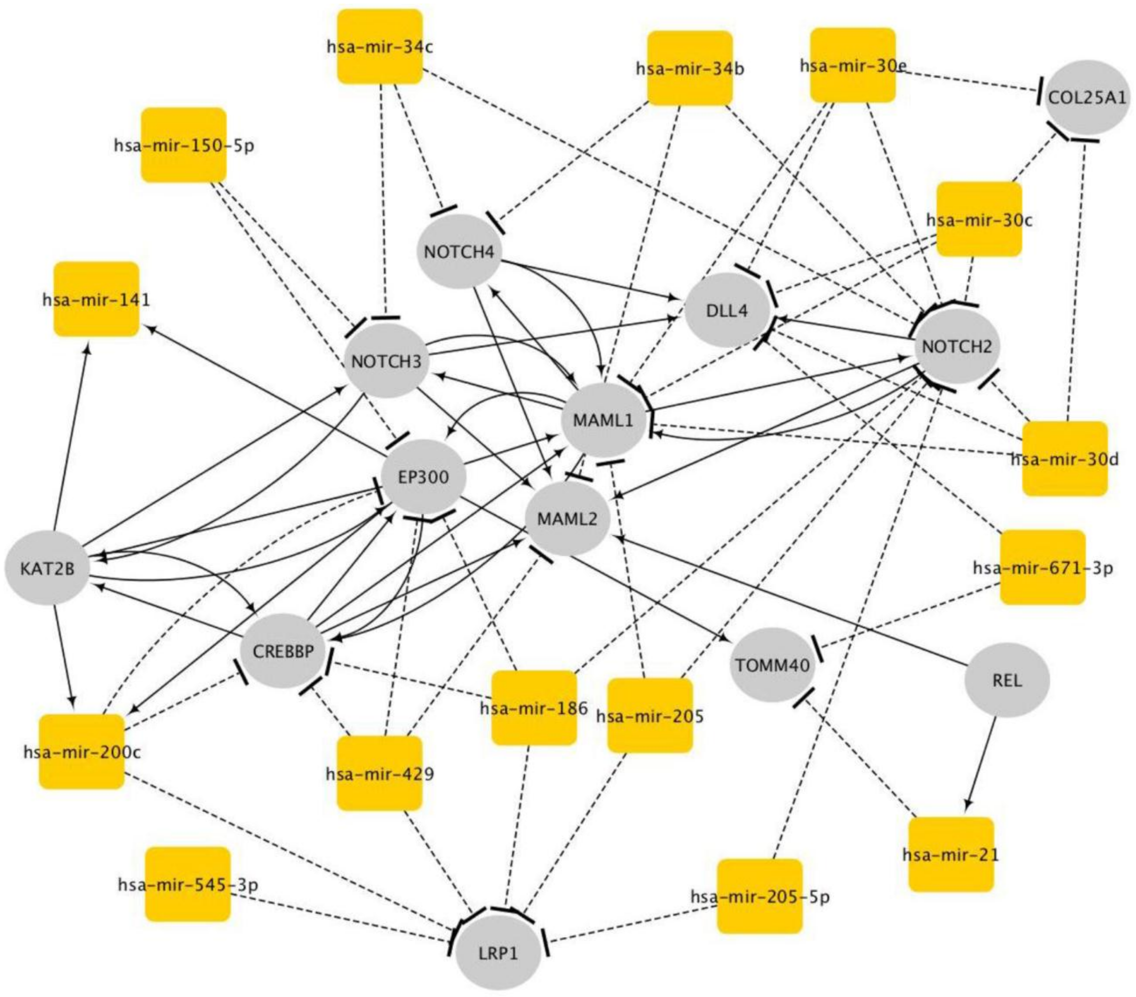


**Supplementary Figure 1. The regulatory interactions between the largest human healthspan pathway and the corresponding enriched miRNAs.**

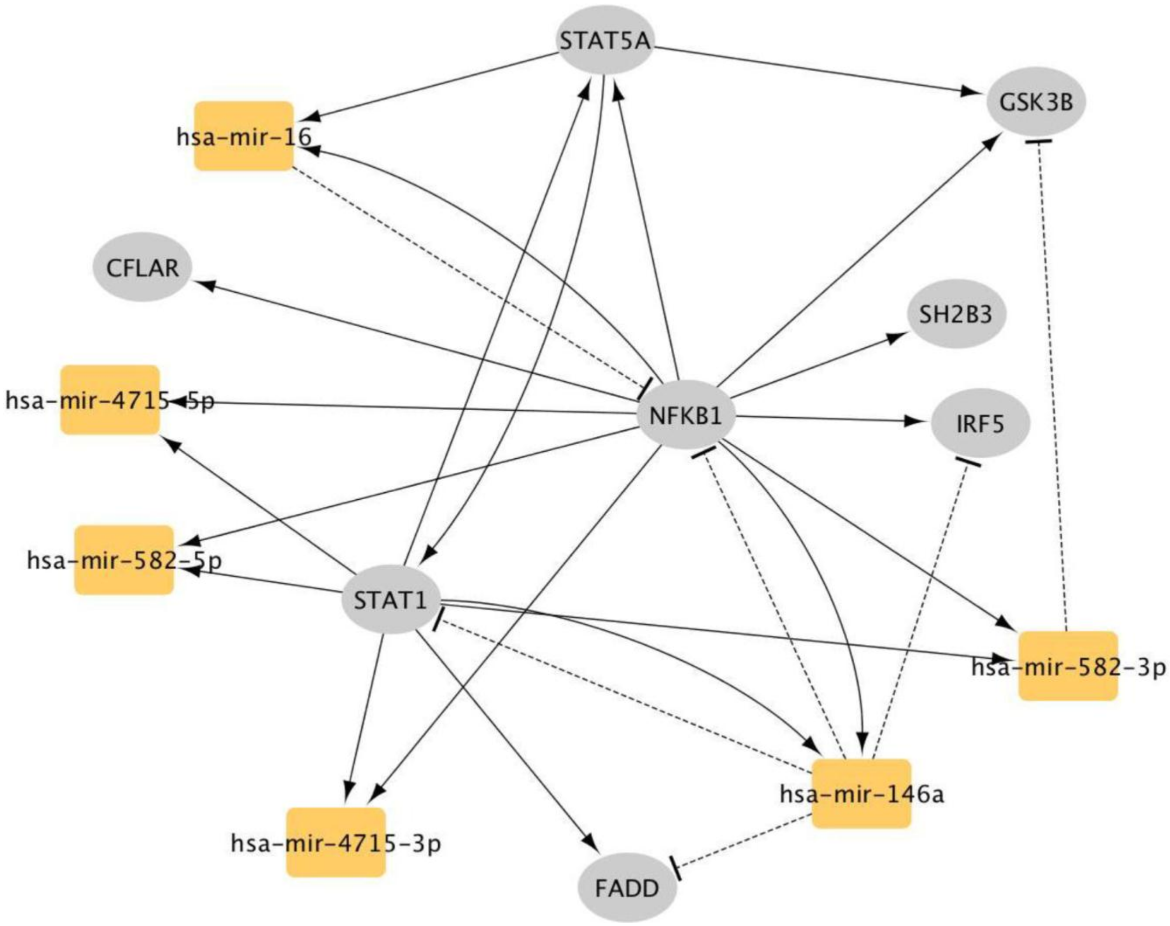


**Supplementary Figure 2. The regulatory interactions between the second-largest hu-man healthspan pathway and the corresponding enriched miRNAs.**

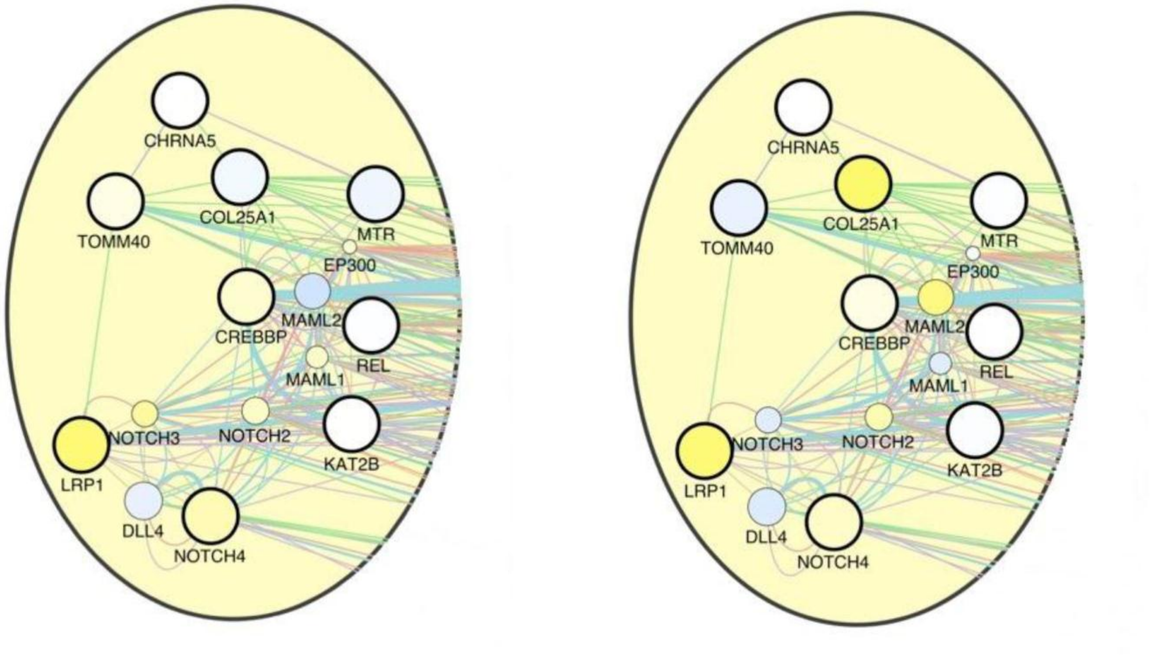


(A) BLOOD (B) SKIN

**Supplementary Figure 3. Comparison of expression patterns in two aging tissues, largest human healthspan pathway.**

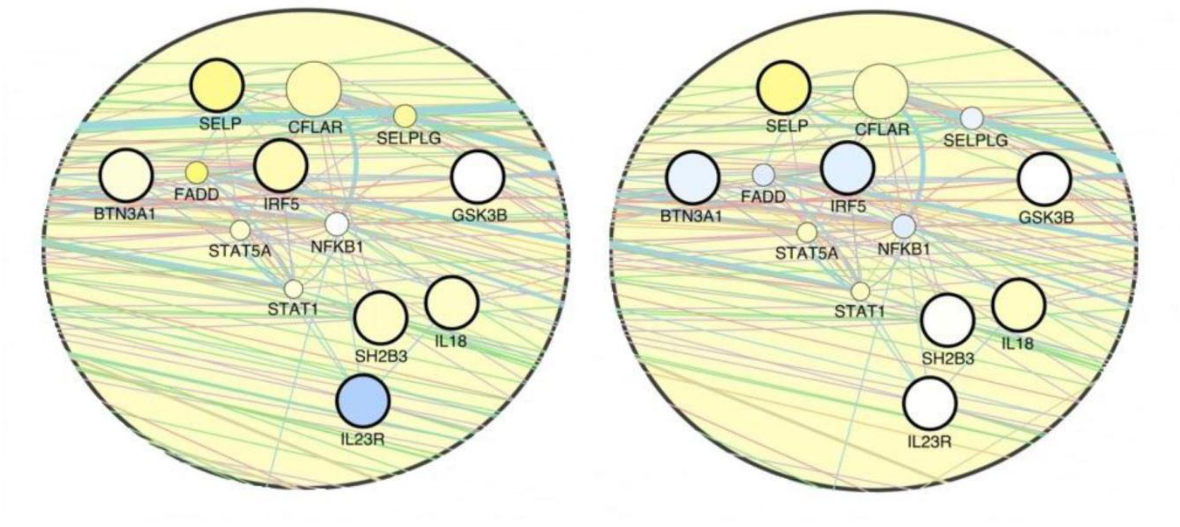


(A) BLOOD (B) SKIN

**Supplementary Figure 4. Comparison of expression patterns in two aging tissues, second-largest human healthspan pathway.**

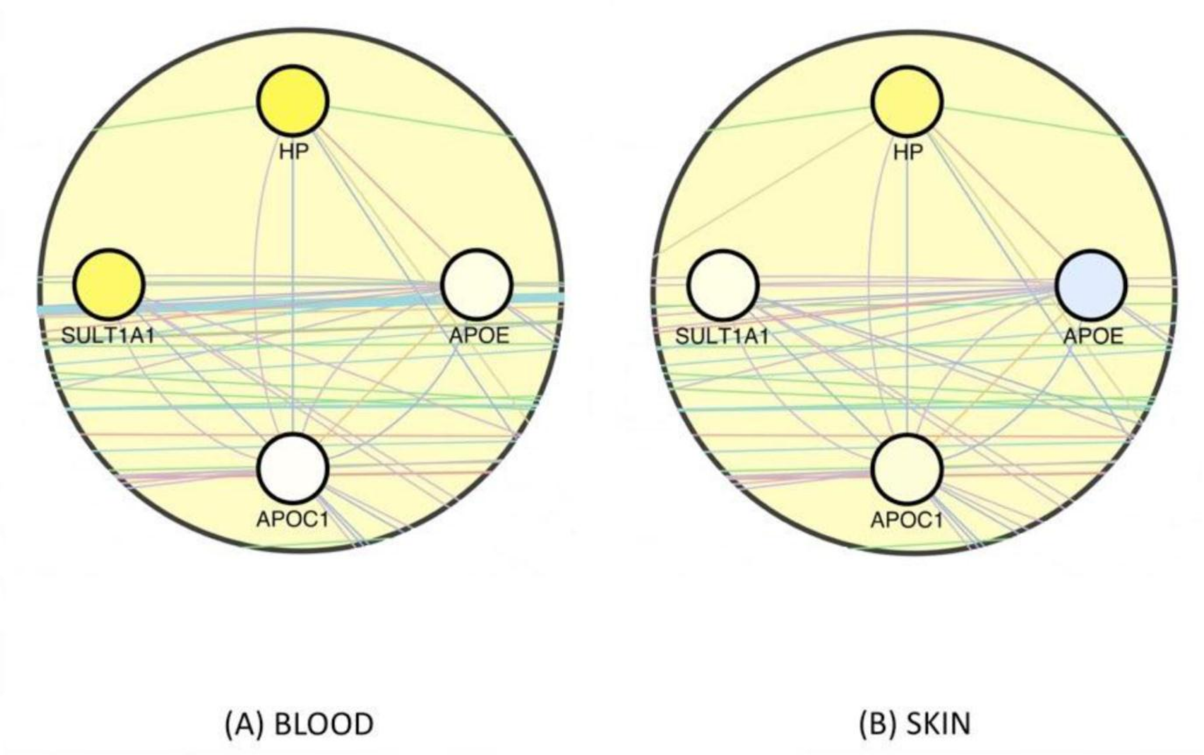


**Supplementary Figure 5. Comparison of expression patterns in two aging tissues, third healthspan pathway.**

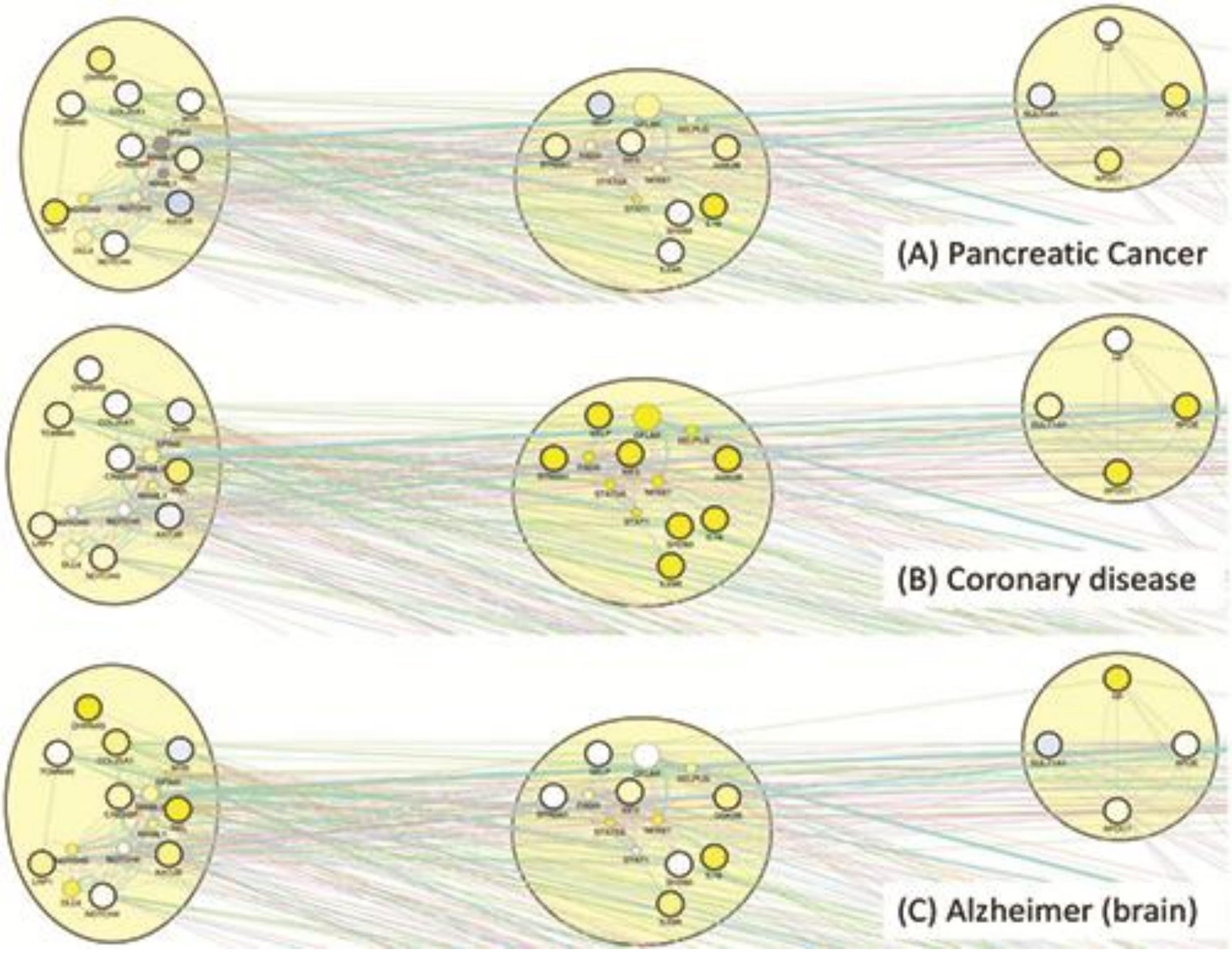


**Supplementary Figure 6. Comparison of expression patterns in three disease-affected tissues, top 3 healthspan pathways.**
