# Supplementary material for: Healthspan pathway maps in C. elegans and humans highlight transcription, proliferation/biosynthesis and lipids
Source: Aging (Albany NY). 2020 Jul 7;12(13):12534–81. doi: 10.18632/aging.103514 (PMC7377848; doi:10.18632/aging.103514)
Supplement: Supplementary Tables [file aging-12-103514-s002..pdf]

SUPPLEMENTARY TABLES

Supplementary Table 1. Features of human health, lack of dysfunction(s), and associated genes.

Feature	Associated gene(s)	Reference	Remarks
Healthy Aging Index, defined by systolic blood pressure, pulmonary vital capacity, creatinine, fasting glucose, and Modified Mini-Mental Status Examination score	ZNF704	[69]	LLFS (Long life family study) cohort, suggestive association nominally replicated in CHS and FHS
Healthy Aging Index	GSK3B	[70]	candidate gene sequencing in LLFS cohort
grip strength, gait speed, physical activity, FEV from exceptional survivors	NBPF6	[71]	LLFS cohort, replicated in Health ABC cohort; phenotype defined based on first principal component of 28 physiologic measurements
lack of frailty	IL-18<br>IL-12A<br>LRP1<br>SELP	[72]	association analysis of 620 polymorphisms with a frailty index (deficit count) in the English Longitudinal Study of Ageing; SNPs did not show genome-wide significance
lack of frailty	MTR<br>CASP8<br>CREBBP<br>KAT2B<br>BTRC	[73]	association analysis of 1354 polymorphisms with a frailty index (deficit count) in Women's Health and Aging Studies I and II; SNPs were not significant genome-wide

Supplementary Table 2. Features of human health, (lack of) multiple diseases, and associated genes.

Feature	Associated gene(s) / pathways	Reference	Remarks
no chronic diseases (by list of ICD codes) and no chronic medications	* BTN1A1, BTN3A1 (MHC locus)<br>* SLC22A4 (→carnitine)<br>* KCNE4<br>* COL25A1 (→amyloid deposition?)	[74] (Wellderly)	whole-genome sequencing; molecular correlate SNPs were not significant genome-wide; sample (e.g., education) bias is likely [75]; no replication was done; COL25A1 was found based on rare variants
candidate SNP identification by disease (atrial fibrillation, cancer, coronary heart disease, diabetes, heart failure, stroke, death) in one study; *candidate confirmation* by the same, and also by other disease (neuronal, vascular, psychiatric and inflammatory), in 5 additional studies	* MAML3<br>* SEMA5B<br>* TCF7L2<br>* ZFHX3<br>* TMPRSS2	[76]	genes based on summary statistics of disease
never diagnosed with cancer, cardiovascular disease, dementia, diabetes or major pulmonary disease	* APOE<br>* HP (haptoglobin)<br>→ lipid and cholesterol maintenance	[77]	targeted genotyping
macular degeneration, multiple sclerosis. menopause onset, rheumatoid arthritis, systemic lupus erythematosus	SLC44A4	[78]	expression QTLs interacting with age implicated in multiple diseases
psoriasis, diabetes	HLA-DQA2		
Alzheimer dementia, cardiovascular disease	* Cholesterol transport endocytosis<br>* Immune response	[79], cf. [80]	overlap of enrichment in GWAS pathway analyses
clustering of 372 disease polymorphisms in the genome	* NOTCH4<br>* CDKN2B (p15INK4b), CDKN2A (p16INK4a/p14ARF), *CDKN2BAS (ANRIL)*<br>* IL23R<br>* REL<br>* TERT<br>* IRF5, TNPO3<br>* IKZF3, GSDMA, GSDMB	[81]	meta-analysis of SNPs in age-associated diseases

Supplementary Table 3. Features of human health, lifespan/longevity mediated by lack of disease, and associated genes.

Feature	Associated gene(s) / pathways	Reference	Remarks
exceptional human longevity, weighted by chronic kidney disease, bone mineral density, LDL, triglycerides, total cholesterol	* APOE, TOMM40 (→Alzheimer's disease), * CDKN2B, *ANRIL* (→cellular senescence), * ABO (→ O blood group), * SH2B3, ATXN2	[82]	diseases/dysfunctions/traits used for weighting selected for their significant genetic overlap with longevity; "many of the SNPs found by iGWAS showed an association for not one, but many diseases which seem to have distinct etiologies"
parental lifespan mediated by participant's risk factors known to be disease-related, particularly education level, LDL, HDL, BMI, smoking, coronary artery disease, diabetes, schizophrenia, height, triglycerides, glucose	* RBM6<br>* SULT1A1<br>* CHRNA5<br>* BSND<br>* CELSR2<br>* TRAIP<br>* C5ORF67<br>* *FTH1P5 (pseudogene)*<br>* LPA<br>* *CDKN2BAS (ANRIL)*<br>* NPIPB8<br>* FTO<br>* APOC1	[83]	based on UK biobank data; using offspring genotypes as proxy for parental genotypes; replication in five independent longevity studies; the paper does not specify which genes are involved in lipid metabolism claimed to be enriched; SNPs in RBM6, SULT1A1, CHRNA5 are "nearby" brain expression QTLs

Supplementary Table 4. Features of *C. elegans* health, based on genetic studies of health, and associated genes.

Feature	Associated gene(s) / pathways	Reference	Remarks
(stimulated) locomotion	hpa-1, hpa-2, let-23, plc-1, itr-1 (→ EGF pathway)	[84]	
	ahr-1	[85]	
	* C15H9.7 (kynu-1) (also Alzheimer pathology delay)<br>* iglr-1<br>* tsp-3 (→ hypoxic response, by pathway interaction)<br>* rcn-1 (rcan-1) (→ mTOR, by pathway interaction)<br>* unc-36 (→ mTOR, by pathway interaction)	[86]	association with stimulated locomotion (*"motivated* movement") and thrashing, based on candidate genes of *C. elegans* orthologs of human genes differentially expressed with age

Supplementary Table 5. Features of *C. elegans* health, based on compound intervention studies affecting health, and associated genes.

Feature	Associated gene(s) / pathways	Reference
stress resistance	**eat-2, skn-1**	cf. [87]
thermal stress resistance	**ahr-1**	[85]
	osr-1, unc-43, sek-1	cf. [87]
	**akt-2, mev-1, nhr-8**	
	**sir-2.1**	
	**age-1, skn- 1, mek-1**	
	**daf-2, daf-16, hsf-1, sod-3, hsp-12.3**	
oxidative stress resistance	**osr-1, unc-43, sek-1**	cf. [87]
	**sir-2.1**	
	**age-1, skn- 1, mek-1**	
	**daf-2, daf-16, hsf-1, sod-3, hsp-12.3**	
	**sek-1, daf-16, eat-2, mev-1**	
	**daf-2, akt-2, mev-1, nhr-8**	cf. [88]
locomotion	* ins-1 (Ins/IGF-1)	cf. [89]
	* pdk-1 (→ pyruvate metabolism)	
	* hsf-1, skn-1 (nrf2)	
	daf-2, daf-16, **hsf-1,** sod-3, hsp-12.3	cf. [87]
	tph-1	
pharyngeal pumping	**ahr-1**	[85]
	akt-2, mev-1, nhr-8	cf. [87]
	sir-2.1	
	**tph-1**	
	**daf-2, daf-16, hsf-1**	
	ets-7	[90]
	age-1, **skn-1,** mek-1	cf. [89]
reproduction	eat-2	cf. [87]
	**tph-1**	
	* **Ins/IGF-1**	cf. [89]
	* **sirtuins**	

Supplementary Table 6. Human genes associated with health, listing all genes from Tables 1–3.

Human gene	GenAge information (Tacutu et al., 2018)
ZNF704	-
GSK3B	Target of genes previously linked to ageing
NBPF6	-
IL-18	-
IL-12A	-
LRP1	-
SELP	-
MTR	-
CASP8	-
CREBBP	Regulation or control of genes previously linked to ageing
KAT2B	-
BTRC	-
BTN1A1	-
BTN3A1	-
SLC22A4	-
KCNE4	-
COL25A1	-
MAML3	-
SEMA5B	-
TCF7L2	-
ZFHX3	-
TMPRSS2	-
APOE	Linked to human longevity and/or multiple age-related phenotypes
HP	-
SLC44A4	-
HLA-DQA2	-
NOTCH4	-
CDKN2B	Directly linked to ageing in a cellular model system
CDKN2A	Directly linked to ageing in a cellular model system
IL23R	-
REL	-
TERT	Directly linked to ageing in a cellular model system
IRF5	-
TNPO3	-
IKZF3	-
GSDMA	-
GSDMB	-
TOMM40	-
ABO	-
SH2B3	-
ATXN2	-
RBM6	-
SULT1A1	-
CHRNA5	-
BSND	-
CELSR2	-

TRAIP	-
C5ORF67	-
LPA	-
NPIPB8	-
FTO	-
APOC1	-

Supplementary Table 7. ***C. elegans*** **genes associated with health, listing all genes from Tables 4, 5.**

C. elegans gene	GenAge information (Tacutu et al., 2018)
hpa-1	Median lifespan is 30% higher in mutants
hpa-2	Median lifespan is 15% higher in mutants
let-23	19% decrease of median lifespan and 8% decrease of maximum lifespan in reduction-of-function mutants; 29% increase of median lifespan and 9% increase of maximum lifespan in gain-of-function mutants
plc-1	-
itr-1	Increased ITR-1 activity extends median and maximum lifespan (53% increase of median lifespan, 29% increase of maximum lifespan); reduced ITR-1 activity shortens culture survival (i.e., -11% increase of median lifespan, -31% increase of maximum lifespan)
ahr-1	-
C15H9.7	-
iglr-1	-
tsp-3	-
rcn-1	-
unc-36	-
ins-1	Increased dosage increases lifespan by 25%
pdk-1	Loss-of-function alleles extend lifespan by 60%
hsf-1	Transgenic overexpression-mutants live longer (median lifespan ~50% higher) and are more thermotolerant; RNAi resulted in a 74% decrease in median lifespan in daf-2 mutant background and a 45% decrease in lifespan in daf-2/daf-16 double mutant background
skn-1	RNA interference or mutations prevented the life-extension effects of dietary restriction; mean lifespan is 5-20% higher after overexpression
daf-2	Mutations double adult lifespan; post developmental RNAi resulted in a 79% increase in mean lifespan
daf-16	Average lifespan is 45% lower by using RNAi
sod-3	-
hsp-12.3	-
tph-1	-
akt-2	-
mev-1	Mutants had a decreased lifespan, are hypersensitive to raised oxygen concentrations, and their lifespan decreases dramatically as oxygen concentrations increase
nhr-8	Median lifespan is up to 35% lower in mutants
sir-2.1	Overexpression extends lifespan uo to 50%; sir-2.1(ok434) mutants show a slight decrease in lifespan as well as sensitivity to various stresses
ets-7	-
age-1	Maximum and average lifespan are up to 10-fold greater in mutants
mek-1	-
eat-2	Mutations result in partial starvation by disrupting the function of the pharynx and an approximately 50% extension of lifespan
osr-1	-
unc-43	-
sek-1	-

Supplementary Table 8. *C. elegans* genes associated with health, listing genes based on WormBase gene expression data.

C. elegans gene	GenAge information (Tacutu et al., 2018)
hop-1	-
frk-1	-
itr-1	Increased ITR-1 activity extends median and maximum lifespan (53% increase of median lifespan, 29% increase of maximum lifespan); reduced ITR-1 activity shortens culture survival (i.e., -11% increase of median lifespan, -31% increase of maximum lifespan)
jun-1	-
sad-1	-
unc-43	-
nhx-2	RNAi led to a loss of fat stores in the intestine and a 40% increase in lifespan
hlh-2	-
rab-11.1	-
mlk-1	-
pept-1	Deletion results in retarded development, reduced body size, and extended reproductive lifespan; it also further extends (60%) the life-extension caused by daf-2 mutations
pig-1	-
rig-6	-
let-767	-
egl-44	-
abts-1	-
elo-2	RNAi resulted in lifespan extension (mean lifespan 9% higher)
daf-22	-
pak-2	-
stn-1	-
mig-10	-
unc-68	-
unc-52	RNAi in adulthood extended mean lifespan by 11%
C35E7.10	-
nhr-8	Median lifespan is up to 35% lower in mutants
gsp-4	-
elo-6	-
gsp-3	-
C18H7.4	-
ilys-3	-
C34F11.5	-
elo-5	RNAi decreased median lifespan by 45% in wild type animals and 29% in daf-2 mutants
elo-4	-
bub-3	6% mean lifespan extension by using RNAi
unc-5	-
pgp-5	-
rhgf-2	-
hbl-1	-
ima-2	-
gsto-1	-
acly-1	-
mtm-6	-

mrg-1	-
rbr-2	rbr-2(tm1231) strain displays reduced longevity; rbr-2(ok2544) strain exhibits longer mean and maximum lifespan, both at 20°C (~14%) and 25°C (~15%); overexpression can extend lifespan of adult wild-type animals at 20°C; mean lifespan is 37% higher by using RNAi
acox-1	-
apl-1	Mean lifespan is 20% higher in overexpression conditions
lev-11	-
cat-4	-
acox-5	-
glr-2	-
ceh-44	-
unc-13	Mutation results in a 150% life-extension in males and 32% in hermaphrodites
haf-4	-
ddr-1	-
eps-8	-
phy-2	-
ZC376.2	-
sor-3	-

Supplementary Table 9. List of diseases and functional terms associated with the miRNAs enriched as regulators of the largest human healthspan pathway.

Associated Terms	miRNAs	Adjusted P-val
Epithelial-mesenchymal transition	hsa-mir-200c, hsa-mir-141, hsa-mir-429, hsa-mir-205, hsa-mir-30c, hsa-mir-30d, hsa-mir-30e, hsa-mir-21	0.0007
Nephrosclerosis	hsa-mir-141, hsa-mir-429, hsa-mir-205	0.0003
Kidney Neoplasms	hsa-mir-21, hsa-mir-200c, hsa-mir-141	0.0005
Lupus Erythematosus, Systemic	hsa-mir-200c, hsa-mir-205, hsa-mir-429, hsa-mir-141, hsa-mir-21	0.0005
Carcinoma, Non-Small-Cell Lung	hsa-mir-205, hsa-mir-21, hsa-mir-30d, hsa-mir-34b, hsa-mir-34c, hsa-mir-200c, hsa-mir-429, hsa-mir-30e, hsa-mir-186	0.0005
Esophagus	hsa-mir-205, hsa-mir-21	0.0006
Intracranial Aneurysm	hsa-mir-34b, hsa-mir-34c	0.0006
Helplessness, Learned	hsa-mir-141, hsa-mir-200c, hsa-mir-429	0.0007
Barrett Esophagus	hsa-mir-21, hsa-mir-200c, hsa-mir-141, hsa-mir-429	0.0011
Neoplasms	hsa-mir-21, hsa-mir-30d, hsa-mir-200c, hsa-mir-141, hsa-mir-429, hsa-mir-30e, hsa-mir-34b, hsa-mir-34c, hsa-mir-205	0.0018
Small Cell Lung Carcinoma	hsa-mir-34b, hsa-mir-34c	0.0019
Melanoma	hsa-mir-30d, hsa-mir-429, hsa-mir-200c, hsa-mir-205, hsa-mir-186, hsa-mir-30e, hsa-mir-21, hsa-mir-34b, hsa-mir-34c, hsa-mir-141	0.0021
Endometrial Neoplasms	hsa-mir-186, hsa-mir-21, hsa-mir-200c, hsa-mir-141, hsa-mir-429, hsa-mir-205	0.0028
Sarcoma	hsa-mir-34b, hsa-mir-34c	0.0037
Mouth Neoplasms	hsa-mir-200c, hsa-mir-141, hsa-mir-21, hsa-mir-205	0.0042
Cholangiocarcinoma	hsa-mir-141, hsa-mir-21, hsa-mir-200c	0.0053
Aortic Aneurysm, Thoracic	hsa-mir-30d, hsa-mir-30e, hsa-mir-21	0.0053
Ovarian Neoplasms	hsa-mir-21, hsa-mir-141, hsa-mir-429, hsa-mir-30d, hsa-mir-200c, hsa-mir-34b, hsa-mir-34c, hsa-mir-30e	0.0078
Aortic Valve Stenosis	hsa-mir-141, hsa-mir-21	0.0089

Adenocarcinoma	hsa-mir-205, hsa-mir-429, hsa-mir-200c, hsa-mir-21, hsa-mir-34b	0.0093
Carcinoma, Renal Cell	hsa-mir-141, hsa-mir-200c, hsa-mir-429, hsa-mir-34b, hsa-mir-34c, hsa-mir-205, hsa-mir-21, hsa-mir-30d	0.0095
Mesothelioma	hsa-mir-21, hsa-mir-30e, hsa-mir-34b, hsa-mir-34c	0.0096
Thyroid Neoplasms	hsa-mir-141, hsa-mir-21, hsa-mir-34b, hsa-mir-30d, hsa-mir-200c	0.0101
Urinary Bladder Neoplasms	hsa-mir-205, hsa-mir-21, hsa-mir-34b, hsa-mir-34c, hsa-mir-429, hsa-mir-200c, hsa-mir-141	0.0103
Heart Failure	hsa-mir-186, hsa-mir-200c, hsa-mir-205, hsa-mir-21, hsa-mir-30e, hsa-mir-34b, hsa-mir-429, hsa-mir-34c	0.0115
Carcinoma, Ehrlich Tumor	hsa-mir-429, hsa-mir-141	0.0123
HBV Infection	hsa-mir-34b, hsa-mir-34c	0.0123
Esophageal Neoplasms	hsa-mir-21, hsa-mir-200c, hsa-mir-141, hsa-mir-205, hsa-mir-34b, hsa-mir-34c	0.0125
Lymphoma, Large B-Cell, Diffuse	hsa-mir-21, hsa-mir-200c	0.0161
Huntington Disease	hsa-mir-34b, hsa-mir-200c	0.0204
Obesity	hsa-mir-21, hsa-mir-30e	0.0204
Carcinoma, Squamous Cell	hsa-mir-21, hsa-mir-205, hsa-mir-30d, hsa-mir-200c, hsa-mir-34b, hsa-mir-34c	0.0217
Leukemia, Promyelocytic, Acute	hsa-mir-34b, hsa-mir-34c	0.0251
Lung Neoplasms	hsa-mir-205, hsa-mir-21, hsa-mir-30d, hsa-mir-30e, hsa-mir-34b, hsa-mir-34c, hsa-mir-186, hsa-mir-200c	0.0267
ACTH-Secreting Pituitary Adenoma	hsa-mir-141, hsa-mir-21	0.0302
Diabetic Nephropathies	hsa-mir-21, hsa-mir-200c	0.0302
Prostatic Neoplasms	hsa-mir-21, hsa-mir-205, hsa-mir-34c, hsa-mir-200c, hsa-mir-141, hsa-mir-34b, hsa-mir-30d	0.0369

Supplementary Table 10. List of diseases associated with the miRNAs enriched as regulators of the second-largest human healthspan pathway.

Associated Terms	miRNAs	Adjusted P-val
Eczema	hsa-mir-146a	0.010
Chlamydia Infections	hsa-mir-146a	0.010
Creutzfeldt-Jakob Syndrome	hsa-mir-146a	0.010
Gerstmann-Straussler-Scheinker Disease	hsa-mir-146a	0.010
Arthritis, Psoriatic	hsa-mir-146a	0.021
Sjogren's Syndrome	hsa-mir-146a	0.021
Moyamoya Disease	hsa-mir-146a	0.021
Myocardial Reperfusion Injury	hsa-mir-146a	0.021
Behcet Syndrome	hsa-mir-146a	0.021
Psychotic Disorders	hsa-mir-146a	0.021
Prion Diseases	hsa-mir-146a	0.031
Influenza, Human	hsa-mir-146a	0.041

Supplementary Table 11. Overlap of healthspan pathway genes: first network alignment.

Human gene		Putative function	Kind of inter-action	GenAge information	Orthologues *C. elegans* gene		Putative function	Kind of inter-action	GenAge information
PAK4	serine/threonine kinase signaling	protects from apoptosis [32]<br>overexpression/hyperstimulation is associated with cancer [34]; [35]; [36]<br>Wnt signaling [91]<br>Cytoskeletal reorganization [92], [93]	shared domains, genetic interaction, and pathway data	none available	<u>*pak-2*</u>	serine/threonine kinase signaling	differentially regulated during ageing l., [94]	shared domains (except for the predicted interaction of gsk-3 and C25G6.3, which is based on the Interologous Interaction Database)	none available
BRSK2		response to DNA damage [95]; [41]<br>neuronal differentiation [43]; [44]<br>regulator of glucose-stimulated insulin secretion [43]			<u>*sad-1*</u>		regulation of neuronal polarization and synapse organization [96]; [97]<br>tau-protein kinase activity *in vitro* [98]		
MELK		regulation of cell cycle [45]<br>involved in apoptosis [47]			<u>*pig-1*</u>		regulation of programmed cell death [99]<br>neuronal development [100]; [101]		
<u>GSK3B</u>		Wnt signaling [102]<br>associated with Alzheimer's and Parkinson's disease [103]; [104]		target of genes previously linked to ageing	*gsk-3*		Wnt signaling [105]<br>Tau phosphorylation [106]<br>apoptotic cell clearance [107]		
<u>CDKN2B</u>		cyclin-dependent kinase inhibitor [108]<br>tumor suppressor via inhibition of cell cycle progression [109]<br>involved in cardiovascular and metabolic diseases [110]		linked to ageing in a cellular model system	*C25G6.3*		increased expression during pathogenic stress [111]		

Underlined genes indicate that gene originates from the original lists of health genes. Not underlined genes are orthologs. Colors are based on gene expression changes triggered by rapamycin (in case of *C. elegans*) or by caloric restriction (in case of human), see Figures 1–3.

Supplementary Table 12. Overlap of healthspan pathway genes: second network alignment.

Human gene	Putative function	Kind of inter-action	GenAge information	Orthologues *C. elegans* gene		Putative function	Kind of inter-action	GenAge information
ACOX1	fatty acid beta-oxidation pathway [112]<br>knockdown results in ROS overproduction [113]	co-expression, co-localization and physical interaction (except for the interaction of SLC5A1 and GCH1, which is genetic)	none available	<u>*acox-1*</u>	differentially regulated in a long-lived *sir-2.1* overexpression strain (Viswanathan and Guarente, 2011) and during osmotic stress (Burton et al., 2017)	biosynthesis of the fatty acid component of dauer pheromones [114]	co-expression	none available
SCP2	intracellular lipid transfer [115]<br>involved in Zellweger syndrome [116]			<u>*daf-22*</u>		biosynthesis of the fatty acid component of dauer pheromones [114]<br>regulation of fat metabolism [117]		
GCH1	affects cardiovascular risk [118]<br>associated with dopamine-responsive dystonia [119]			<u>*cat-4*</u>		dopamine biosynthetic processes [120] (<br>target of the stress-related transcription regulator *skn-1* (Oliveira et al., 2009).		
SLC15A1	overexpressed in human cancer cell lines [121]<br>nutrient transport processes [122]			<u>*pept-1*</u>		involved in insulin and TOR signalling [123]<br>induced transcription in healthspan-promoting treatments (Ihara et al., 2017; Cai et al., 2014; Pietsch et al., 2012)		Deletion results in extended lifespan and reproductive lifespan

Underlined genes indicate that gene originates from the original lists of health genes. Not underlined genes are orthologs. Colors are based on gene expression changes triggered by rapamycin (in case of *C. elegans*) or by caloric restriction (in case of human), see Figures 1–3.
